# Antitrypsin deficiency: still more to learn about the lung after 60 years

**DOI:** 10.1183/23120541.00139-2024

**Published:** 2024-07-22

**Authors:** Robert A. Stockley, David G. Parr

**Affiliations:** 1 Lung Investigation Unit, Medicine – University Hospitals Birmingham NHS Foundation Trust, Queen Elizabeth Hospital Birmingham, Edgbaston, UK; 2 Respiratory Medicine, University Hospitals Coventry and Warwickshire NHS Trust, Coventry, UK

## Abstract

The past 60 years have seen multiple publications related to lung disease in α_1_-antitrypsin deficiency largely reflecting the pathophysiology, biochemical effect and outcomes of augmentation therapy. However, the complexity of disease phenotype and the impact of the natural history presents problems of patient management, study design and hence interpretation of outcome. Although many national and some international registries exist, the lack of consistent in-depth assessment and importantly, the impact of augmentation therapy likely influences our perception of the true natural history.

Development of new therapeutic strategies, and even assessment of the role and efficacy of augmentation, remain a challenge as powering such studies for conventional COPD outcomes is impractical due to relative rarity of the genetic condition and the presence of clinical phenotypic variation.

The current review approaches these issues, discusses the nature and complexity of assessing patient variability, and provides guidance on further studies required to address them.

## Shareable abstract

**Recent years have explored the natural history and pathophysiology of α_1_-antitrypsin deficiency in more detail including the partial response to augmentation therapy. This review expands on clinical risk, phenotype and therapeutic efficacy.** https://bit.ly/3J4GNJy

## Introduction

60 years have passed since the chance finding of α_1_-antitrypsin deficiency (AATD) as an unexpected biochemical oddity on a protein electrophoretic strip. The association with emphysema was quickly recognised, and a picture was constructed of its relationship to severe, early-onset of lung disease in patients with a limited smoking history, and a clinical phenotype that was distinct from non-deficient COPD [1]. It has since become widely accepted that the predisposition to the development of basal, panlobular emphysema, as opposed to the apical centrilobular emphysema characteristic of usual COPD, is consequent to an imbalance between serine proteinases (generally considered to be neutrophil elastase) and impaired control of tissue damage and pulmonary inflammation arising from deficiency of α_1_-antitrypsin (AAT). In essence, proteolytic destruction of lung elastin leads to emphysema due to impaired physiological control by a suboptimal amount and function of circulating and lung AAT. On the basis that this explanation of emphysema pathogenesis in AATD reflects the complete picture, correction of deficiency with therapeutic augmentation of AAT should prevent the progression of emphysema. Unfortunately, this does not seem to be the reality, which raises significant doubt on the validity of this hypothetical mechanism as the complete picture of the pathogenesis of AATD lung disease.

Neutrophilic inflammation is believed to be central to the pathogenesis of emphysema and to be the principal driver of lung tissue damage. Neutrophil migration into and through lung tissue in response to inflammatory stimuli is associated with the release of high concentrations of serine proteinases, in particular neutrophil elastase (NE). The consequent proteolysis is essential to permit neutrophil penetration between endothelial cells and through the interstitium, and necessitates the destruction of connective tissue, including elastin fibres. This process is permitted but, importantly, limited by inhibitors, notably AAT, in order to restrict the extent of tissue destruction. The relative concentrations of both enzymes and inhibitors determine what might be viewed as “physiological levels” of tissue destruction [2]. *In vitro* studies of inhibitor control of this neutrophilic damage indicate that it can largely be contained in the presence of ∼7 μM AAT [3]. In AATD, this control is compromised by several factors including the reduced inhibitory function arising from low tissue concentrations of AAT [4] and the accumulation and local activation of neutrophils at sites in the lung where AAT polymers have become deposited [5]. Progressive elastin degradation compromises alveolar integrity resulting in the development of emphysema and, when this is associated with airflow limitation, COPD.

Severe deficiency, which is most often seen in the ZZ genetic variant, increases the risk of developing emphysema even in the absence of smoking. In contrast, heterozygotes, such as the SZ and MZ variants in whom the plasma AAT concentration largely exceeds 10 μM, do not appear at risk of developing COPD under these circumstances [6, 7]. This would suggest that there is a “protective level” for plasma AAT, and this putative protective threshold forms the base target for AAT augmentation therapy that was established in the early 1980s [8] and which continues to be used worldwide. Inherent in this theory was the belief that emphysema arising from AAT deficiency could be prevented through AAT augmentation above the “protective threshold”. Proving this in a definitive clinical trial was not deemed possible for a number of reasons relating to the rarity of the disease and the limitations of using the measure that had become inextricably linked with monitoring disease progression, the forced expiratory volume in 1 s (FEV_1_), as the primary outcome [9].

Although several national and international registries for AATD were developed describing progression (largely based on FEV_1_ decline), disease treatment and mortality, there remains little detailed study of the untreated natural history of AATD. However, in recent years more focussed studies of augmentation therapy, the level of risk in heterozygotes and the development and progression of untreated subjects have emerged. The current review explores these data, questioning our inferred concepts of the disease and its management, and offering a potential template for future studies of the natural history, its clinical impact and development of therapeutic strategies.

## The clinical phenotype and natural history of AATD lung disease

In-depth understanding of clinical phenotype (plus the source of phenotypic variability) and natural history are fundamental to the understanding of clinical management, including therapeutic decision-making and prognostication, and to inform drug development programmes. In particular, knowledge of the different disease stages and disease continuum (which may also influence clinical phenotype), the risk of disease progression and the type of monitoring appropriate for different disease stages and clinical phenotypes is essential. Nevertheless, collection of sufficient, high-quality data in order to generate this kind of detail is challenging in a rare disease, particularly one for which putative disease-modifying therapy is widely utilised. As with non-deficient COPD, the development, progression and severity of disease in AATD has largely been defined by the use of the FEV_1_ with a natural history assumed to be one of slow steady decline as depicted, for example, in the Fletcher and Peto curves [10]. However, this assumption is now being challenged by data derived from registries in countries especially where patients do not have ready access to disease-modification from augmentation therapy.

## The belief that the development of emphysema follows a defined path

**FIGURE 1:**
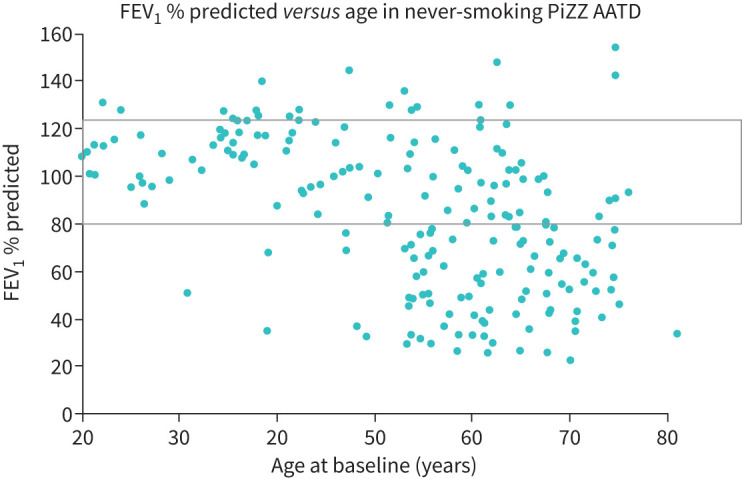
Data show the baseline FEV_1_ expressed as a % predicted for the never-smoking patients in the UK registry related to their age at recruitment. Each point represents an individual patient. FEV_1_: forced expiratory volume in 1 s.

In the classical studies by Hogg and colleagues, pathological evidence had shown that the initial phase of COPD and emphysema development involved the loss of small airways. This not only preceded progressive decline in FEV_1_ [11] but was shown to be extensive before development of impaired FEV_1_. The development of lung function impairment and FEV_1_ decline in young adults with AATD was largely unknown but is now becoming clearer. The initial Swedish “cross-sectional” study demonstrated that young patients had relatively preserved FEV_1_, but this was progressively reduced with age [12]. These data are comparable with that seen in the UK database of non-augmented patients, as shown in figure 1. The UK cohort has also shown that the initial small airway changes described in usual COPD also occur in the early stages of AATD-associated lung disease: a proportion of younger patients have clear physiological evidence of small airways dysfunction (SAD) and symptoms, whilst the FEV_1_ remains in the normal range and before emphysema is radiologically evident [13]. However, these subjects were shown to subsequently develop FEV_1_ decline, whereas it remained stable in those without SAD. SAD therefore also provides an early marker for the development of COPD in AATD and, potentially, an early window of opportunity to modify the pathophysiology before extensive, irreversible disease becomes established. Understanding this earlier stage of disease is essential and should provide a clear target for future preventative strategies.

AATD is classically associated with an emphysematous clinical phenotype leading to a combination of progressive impairment of FEV_1_ and gas transfer. This arises from a variable combination of loss of elastin support of the small airways causing dynamic airways collapse plus the loss of alveolar integrity, which will also impair gas transfer. However, impairment of these physiological measures, although generally correlated, is not always concordant in large studies, with some individual patients having “normal” FEV_1_ but impaired gas transfer and others demonstrating the reverse pattern [14–16]. Computed tomography (CT) lung densitometry indicates that this physiological discordance reflects the apico-basal distribution of emphysema: the FEV_1_ and other measures of airflow obstruction are more reflective of the degree of basal emphysema whereas gas transfer and other measures of gas exchange are more reflective of apical disease [14]. This difference could reflect the influence of gravity on ventilation perfusion matching and on airways collapse in the conventional sitting position for lung function assessment. Alternatively, it may represent differences in the pattern of physiological impairment arising from emphysema pathological sub-type; namely, apical centrilobular and basal panlobular emphysema [14].

Whatever the explanation for these patterns, they illustrate significant heterogeneity in AATD-associated lung disease and represent distinct emphysema phenotypes. Firstly, the classical basal panlobular distribution with a more dominant effect on airflow obstruction and secondly an upper zone centrilobular distribution (similar to non-deficient COPD) that occurs in up to 30% of AATD patients [14] with a greater impact on gas exchange. A functional genetic polymorphism of MMP 9 has been implicated in upper zone emphysema in non-deficient COPD [17], and a single study in AATD indicated an increased prevalence of MMP genetic polymorphisms related to gas transfer [18]. This suggests a possible pathophysiological link and that this polymorphism may act as a genetic modifier to the classical PiZZ-associated clinical phenotype of basal panlobular emphysema and is worthy of further study.

However, two subsequent studies have added some further insight into the pathway of emphysema development and progression. Firstly, a sibling-paired study [19] compared the index sibling (those diagnosed because of symptom presentation) with their non-index (but ZZ) sibling identified by family screening. The index sibling had worse lung function thought to reflect the more intrusive symptomatology that would have led to earlier clinical presentation and diagnosis. The absolute FEV_1_ values (and the severity of emphysema in the lower lung) showed no correlation between siblings whereas gas transfer (and the severity of upper lung emphysema) did. Because the non-index siblings were generally younger than the index sibling, it was considered that this sibling disparity could reflect the pathobiological development of AATD lung disease. In addition, it implied that upper lung abnormality occurred earlier than lower lung abnormality. To investigate this further a second analysis of never-smokers with AATD was undertaken, and physiological development compared to age was tracked using logistic regression to identify when physiological deviation from the normal range was likely to have occurred (*i.e.* when group data were persistently below 100% predicted for age, sex and height). This analysis demonstrated that gas transfer started to deviate late in the third decade, whereas FEV_1_ remained generally normal until early in the fifth decade [20]. In view of this early change in gas transfer, age-related health status (St George's Respiratory Questionnaire (SGRQ)) and lung density in the upper and lower zones were also tracked. The SGRQ data were already abnormal (suggesting an impact on health) in the late 20s together with a loss of density in the upper zones again linking zonal change to physiology as early features in AATD.

The aforementioned accumulation of data has been derived from a large (>1000 PiZZ patients) data base predominantly consisting of patients who had been detected after presentation with symptoms (index cases), a smaller proportion of siblings identified through subsequent family screening (non-index cases) and a significant number of never-smokers. As such it represents the overall natural progression of PiZZ subjects identified from symptoms and their immediate relatives. However, these data reflect the natural history of the identified cohort of non-augmented patients living in the UK and may not necessarily reflect the natural history of all PiZZ individuals (especially as many PiZZ individuals remain undiagnosed). Comparison to data for non-augmented individuals is, therefore, advised as even in cohorts where augmentation is available, factors influencing therapeutic decisions (for example, high symptom load, low physiology, presumed rapid decline and age) may bias group demographics.

In 1972–1974, neonatal testing of Swedish newborns identified 129 PiZZ subjects who have been followed cross-sectionally to the current age of 42 years when 99 of the surviving patients were available for testing [21]. Of these, the majority were never-smokers, and those who had smoked had a limited smoking history. Lung function was obtained from local hospitals for 67 patients, and although most had lung function in the normal range, nine had an FEV_1_/forced vital capacity ratio <0.7 consistent with COPD, of whom five were ex-smokers. Although median gas transfer was in the normal range, some clearly had significantly reduced values consistent with emphysema. The authors concluded that ever-smokers and some never-smokers had “early” physiological evidence of emphysema in the fifth decade consistent with our retrospective study [20]. Unfortunately, no longitudinal data for individuals were reported and hence will not have detected individuals with physiological progression above that of normal ageing, even though their values remain within the “normal range” (as would be expected in early stages of the pathological process).

The consistent descriptions of distinct emphysema phenotypes with specific imaging and physiological characteristics does indicate that there is more than one pathway for the development of chronic lung disease in AATD. It is unclear whether these sub-populations differ in disease progression rates and prognosis, whether they are associated with distinct biomarker profiles and whether clinical outcome measures should be tailored accordingly. However, greater clarity will require more longitudinal data, especially early in the disease process.

## The presence and clinical relevance of bronchiectasis

Bronchiectatic change has become a recognised feature of non-deficient COPD and is associated with bacterial colonisation, increased exacerbation rates and increased mortality [22–24]. The presence of bronchiectasis has also been described in both clinical and pathological studies of AATD. In his early publication, Eriksson [25] described this clinical phenotype in 10% of the initial cohort, and isolated case reports followed [26] together with analysed cohorts [27–29]. Subsequently, it has become usual clinical practice to test bronchiectatic patients for AATD, but a recent study, based on specialist bronchiectasis clinics, identified only a few AATD patients with routine screening and questioned the usefulness of such testing [30].

The establishment of a European AATD data base, EARCO (European Antitrypsin Research Collaborative), enabled this association to be explored further to determine the proportion of AATD patients who presented with bronchiectasis alone. In the initial analysis of over 800 patients with ZZ deficiency, of whom almost 50% had radiological reports of CT scans available, 9% of the patients had radiological features of bronchiectasis alone with little impairment of lung function and most were female with minimal or no smoking history, which may partly explain the lack of emphysema [31].

However, an additional 27% of those with established emphysema also had radiological evidence of bronchiectasis, which is similar to a more detailed previous study [27], and to studies in non-deficient COPD [32]. Recent studies in non-deficient COPD patients with bronchiectasis have highlighted increased exacerbation history [23] and mortality [24]. Whether bronchiectasis alone or combined with emphysema has similar clinical and treatment implications in AATD remains unknown but, in general, Global Initiative for Chronic Obstructive Lung Disease (GOLD) describes associations/comorbidities with COPD as requiring treatment in their own right [33]. Consequently, AATD patients with bronchiectasis may benefit from joint clinics, more extensive characterisation and more intensive intervention.

It is clear that bronchiectasis alone is a feature of a proportion of patients with PiZZ AATD and is similar to the prevalence in specialist bronchiectasis clinics described by Carreto *et al.* [30]. This suggests that AATD testing in bronchiectasis should be continued as this may affect management and inform family screening. Although this association likely represents a further clinical phenotype, it is unclear whether clinical management should differ from non-bronchiectatic AATD patients or non-deficient bronchiectasis. In order to determine similarities to non-deficient bronchiectasis and hence management, AATD bronchiectatic patients require much more individual characterisation, including identification of bacterial colonisation and airways neutrophilia (and serine proteinase activity), as well as exacerbation history and its relationship to physiological decline and emphysema progression.

Non-deficient COPD patients with bronchiectasis are known to have increased bacterial colonisation, recurrent exacerbations and increased mortality as mentioned above. It not only seems likely that this will be similar in AATD COPD patients with bronchiectasis but may even represent a greater inflammatory burden as seen in AATD exacerbations [34] and be amenable (at least in part) to AAT augmentation intravenously [35], by the inhaled route [36] or with more recent oral antiproteinase strategies in development for non-deficient bronchiectasis [37].

The co-localisation of bronchiectatic change and emphysema raises the issue of cause and effect. Certainly, an intensive bronchiectasis management plan aimed at resolving bacterial colonisation and reducing acute exacerbations may have a beneficial effect on local airways damage and, potentially, on co-localised emphysema. The use of inhaled AAT may prove more beneficial than intravenous augmentation therapy in AATD bronchiectasis by influencing local airways inflammation and proteinase activity more efficiently than when given by the intravenous route, as seen in the use of inhaled AAT treatment of cystic fibrosis-associated bronchiectasis [38].

Systemic treatment with Cathepsin C inhibition aimed at reducing pulmonary NE load has been shown to be beneficial in non-deficient bronchiectasis [37]. It is probable, therefore, that this treatment may have a dual benefit on the role of NE in the pathophysiology of emphysema and bronchiectasis in AATD by at least partly restoring an AAT/NE balance in all lung compartments. Clarification of these issues will require better characterisation of AATD patients with bronchiectasis and the instigation of a structured programme of pharmacological and clinical study.

## On the use of augmentation therapy

Augmentation therapy with regular infusions of purified human AAT was a logical development in the 1980s aimed at increasing blood and lung AAT in the understanding that it would provide better local protection against proteolytic damage for those with deficiency. The biochemical case for treatment was proven [8] and subsequent evidence of a reduction in potentially destructive airway inflammation was reported [4]. The treatment became widely accepted for those with emphysema and physiological evidence of lung disease (namely reduced FEV_1_). Since the early changes and outcomes were unknown, the decision to treat was made at the point of clinical presentation in never-smokers or confirmed ex-smokers.

The National Institutes of Health (NIH) registry was established for the purpose of monitoring the clinical effect of this new augmentation, and longitudinal data demonstrated a significant reduction in the subsequent FEV_1_ decline but only in part of the cohort with baseline values between 35% and 49% predicted [39]. Subsequent data provided evidence that FEV_1_ decline influenced mortality [40] and that untreated patients in the registry [39] had greater mortality (although other socioeconomic factors may have played a role). These data provided supportive clinical evidence of the benefit of augmentation that became accepted practice, despite many remaining unanswered questions (see later).

## The decision of when patients should be commenced on treatment

**FIGURE 2:**
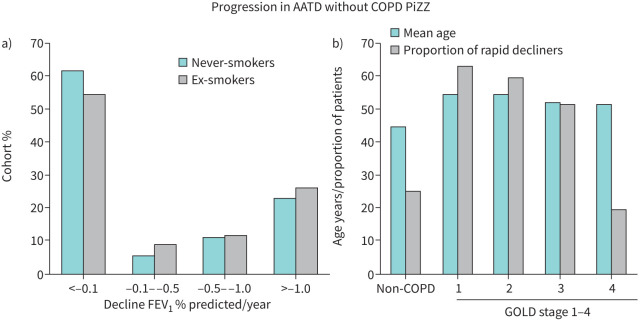
a) The proportion of never- and ex-smokers without established COPD related to their rate of decline in FEV_1_ expressed as % predicted/year. Data derived from UK data base [42, 48]. b) The proportion (%) of PiZZ patients who have a “rapid” decline in FEV_1_ together with the mean age of each group defined by their severity of airflow obstruction as determined by the Global Initiative for Chronic Obstructive Lung Disease (GOLD) staging. FEV_1_: forced expiratory volume in 1 s.

The initial objective decision for treatment is mainly dependent on baseline spirometry in confirmed former smokers or an assumed pretreatment decline in never-smokers rather than pretreatment monitoring. The potential importance of the role of pretreatment monitoring is highlighted by longitudinal data in largely index patients and ex-smokers over up to 15 years [41]. These subjects show variable rates of decline in FEV_1_ and gas transfer from “none” (*i.e.* changes consistent with only age-related decline), to “rapid” (>1% predicted per year) as shown in figure 2a for FEV_1_ in patients without established COPD and figure 2b for those with established COPD.

**FIGURE 3:**
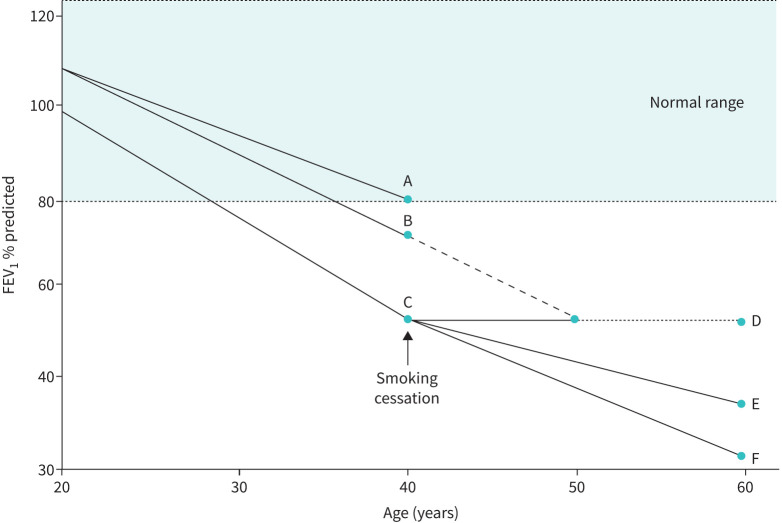
This theoretical figure clarifies the text for interpreting the lung function data at diagnosis. Patient A may have lung function in the normal range despite progression prior to diagnosis. Patient B has more rapid progression compared to patient A but still only mild impairment, but may go on to develop greater impairment if progression occurs in a never-smoker or is not altered by smoking cessation. Patient C presenting with moderate impairment at diagnosis may stabilise with smoking cessation progressing to point D. Alternatively progression may be slowed down by smoking cessation progressing to point E or not be altered progressing to point F. FEV_1_: forced expiratory volume in 1 s.

The existence of this variability will impact on the interpretation of initial post-diagnosis data, which will be dependent on the age of diagnosis and events that preceded this point (especially in former smokers). Abnormal results may be assumed to reflect the progression from an original value somewhere within the “normal” range. However, bearing in mind that the normal range is wide, this raises the issue of what the original normal value was for the diagnosed individual. For example, a 40-year-old presenting with an FEV_1_ of 80% predicted may truly be “normal” with no lung damage but equally may have declined from 110% predicted reflecting a 30% predicted decline (1.5% per year) since the age of ∼20 years. Equally a patient presenting with a value of 70% predicted may only reflect a gradual change if the baseline value was 80% predicted, indicating only a 0.5% predicted decline per year since 20 years of age. Alternatively, it could represent an even more rapid decline if the original baseline value had been 110% predicted, which would indicate a 2% predicted decline per year. This concept is shown graphically in figure 3. Consequently, a snapshot of lung function cannot be used to estimate reliably the rate of progression of lung damage using an assumption of an individual's baseline measurements. Furthermore, physiological measurements (such as FEV_1_) within the normal range may occur in the presence of significant emphysema demonstrable on CT imaging. What may be more important in decision-making is objective evidence that the patient is progressing, especially when this is demonstrably rapid. How this progression is best identified remains contentious. Nevertheless, CT imaging provides more unequivocal evidence of the presence of emphysema than lung function measurements, and data indicate that CT lung density decline is evident across the disease severity spectrum [42] even when FEV_1_ and gas transfer decline are discordant, as discussed below.

## The importance of monitoring to determine treatment

Demonstration of progressive rather than stable disease informs on the potential need to initiate therapy that will retard or, ideally, halt progression. The interpretation of a presumed trajectory of disease progression in AATD is confounded by the influence of tobacco smoke exposure and is more complicated in former smokers than never-smokers, especially if they cease smoking before, at, or after diagnosis (as is almost universal). For example, a cross-sectional comparison of average lung function at different decades suggested different rates of decline and the concept of “slow decliners”. This differential was most evident in the subset of individuals >60 years old [43]. It was noted that in each decade (4th, 5th and 6th) the three “decliner” groups identified had stopped smoking at about the same age (40 s) yet had similar cross-sectional lung function when assessed in the study. The interpretation suggested differential long-term rates of decline determined linearly by age. However, an alternative explanation is that all groups showed the same initial decline which stabilised after smoking cessation, rather than the older cohort showing an overall slower rate of decline than the younger cohorts [43, 44]. Thus, the deleterious effects of cigarette smoking and the benefits of quitting would seem to have a major effect on both early and late disease. Whereas the physiology at diagnosis already has an impact on health status, it does not permit an accurate determination of either previous or likely future progression. Current rate of decline should, therefore, be determined (where possible) by observation from diagnosis rather than linear extrapolation from an assumed normal value and may require annual measurements for up to 3 years for certainty [41].

The aim of augmentation is to slow down and, ideally, prevent the decline in lung function and health. Thus, it could be argued that such therapy should not be indicated in the presence of stability! The importance of this concept is illustrated by longitudinal data from non-augmented patients in the UK data base [41]. Never-smokers show variable physiological progression with >50% without COPD having little evidence of excessive age-related decline and only 25.3% having evidence of rapid progression of FEV_1_ determined as >1% predicted/year. The proportion of rapid decliners varies with disease severity as determined by the FEV_1_ (see figure 2b) although many stabilise. Never-smokers who had lung function consistent with COPD were the minority (<20%), and the prevalence of ex-smokers was greatest with increasing severity at baseline. Of these ex-smokers, 23% showed no subsequent decline when adjusted for age, again demonstrating the benefit of smoking cessation. However, rapid decline (>1% predicted/year) still occurred in >50% of ex-smokers, although this proportion reduced with greater physiological impairment at baseline. This reduction may, in part, reflect a survivor phenomenon with mortality in continued rapid decliners being more likely. Nevertheless, the remainder of those with established COPD (49%), whether never-smokers or ex-smokers, showed minimal progression of FEV_1_ decline beyond normal aging (<1% predicted/year). These observations have implications for decisions on whether or when to start augmentation therapy [41] but should be considered in the context of the following section.

## Is it beneficial to monitor gas transfer in addition to the monitoring of FEV_1_?

**FIGURE 4:**
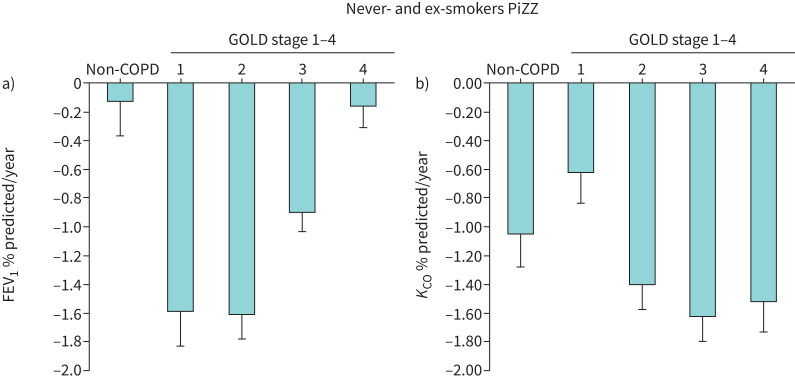
The mean annual decline in a) FEV_1_ and b) gas transfer (*K*_CO_) together with se bar lines is shown related to severity as defined by Global Initiative for Chronic Obstructive Lung Disease (GOLD) for never- and ex-smokers from recruitment. Data are mean with se bar and derived from Stockley *et al*. [46]. FEV_1_: forced expiratory volume in 1 s; *K*_CO_: transfer coefficient of the lung for carbon monoxide.

Transfer coefficient of the lung for carbon monoxide (*K*_CO_) (transfer factor of the lung for carbon monoxide (*D*_LCO_)/alveolar volume (*V*_A_)) is a more accurate physiological test of pulmonary capillary and alveolar function than spirometry, and correlates well with histopathological measures of emphysema and with radiological lung density [45]. Although *K*_CO_ also generally correlates well with FEV_1_, airflow obstruction is a poorer surrogate for monitoring emphysema severity and progression, despite its adopted pivotal role and acceptance as a diagnostic marker of COPD [32]. Importantly these two physiological measures are not always concordant, as discussed above. This discordance is not only evident in cross-sectional analyses but also applies to physiological progression evident in longitudinal data: *K*_CO_ and FEV_1_ can progress independently in some patients [46]. As described above in relation to FEV_1_, the existence of stable and fast decliners is also seen in longitudinal data of gas transfer. Notwithstanding this heterogeneity in individual data, group data indicate that *K*_CO_ decline tends to be most rapid in patients with severe COPD (when defined by FEV_1_). This pattern of *K*_CO_ decline differs from that of FEV_1_ decline, which is most rapid in mid-disease severity defined by GOLD [32], as summarised in figure 4a and b (data derived from Stockley *et al*. [41] and Dawkins *et al.* [47]). Consequently, there is also a case for the recommendation of augmentation therapy for those with rapidly declining *K*_CO_ (or even documented decline in CT lung density) irrespective of the FEV_1_ and its decline. However, in the absence of clinical trials that also include patients with more severe COPD categories, such an approach currently remains conjectural.

The role of other measures of physiological impairment and symptomatology remain features of COPD and its progression. Whether features such as increasing total lung capacity (TLC), air trapping (residual volume (RV) and RV/TLC), objective exercise testing and symptom quantification (SGRQ and COPD Assessment Test) can help define the need for augmentation either alone or as a composite score remains to be explored. However, because of variability of all these measures and their progression, a comprehensive and repeated (for 3 or more consecutive years) or a combination of scores may be necessary to determine trajectory, need for therapy and confirmation of subsequent stabilisation.

## The question of whether augmentation therapy works

Evidence that augmentation therapy works is based on biochemical studies [8], data from the NIH registry [39], “small” interventional studies [48, 49] and other registry observational data [50]. Pulmonary disease in AATD is slowly progressive, and conventional physiology and health status outcomes cannot, therefore, be powered to demonstrate an effect because of the rarity of the disease although careful patient selection may help [51]. Augmentation would be expected (if effective) to slow progression below the normal aging process, reduce the inflammatory response and have a beneficial effect on the nature of exacerbations [52] rather than preventing such episodes. Health status and physiological decline are both highly variable, nonspecific and insensitive measures although also related to lung density [53]. For this reason, lung density measured by CT scanning has been extensively validated being both specific and sensitive to modification of pathological emphysema progression, thereby enabling studies to be powered with this as the outcome.

CT densitometry relates to FEV_1_ (in both cross-sectional and longitudinal studies), gas transfer, health status (in both cross-sectional and longitudinal studies), exercise capacity and exacerbation characteristics, and, importantly, is the best predictor of mortality in AATD [54]. For this reason, several placebo-controlled studies have now been carried out indicating a significant benefit on CT evidence of emphysema progression although not completely halting density decline [55–58]. Since these studies have been designed to provide circulating AAT levels above the “protective” threshold, the failure to prevent emphysema progression completely has raised several possibilities that need addressing:

1. Is the protective threshold higher than has been assumed from biochemical studies and risk observation in heterozygotes?
2. Is there an alternative/additional pathogenetic pathway and/or a non-serine proteinase that also play a role?
3. Is the protective anti-inflammatory role of purified AAT partly offset by the proinflammatory role of the ambient Z protein and accumulation of Z protein polymers in the lung?
4. Is weight-determined dosage appropriate for all?

There are reasons to consider all four as relevant. Firstly, although the presence of a protective threshold has been supported by *in vitro* studies and observations of heterozygote risk *in vivo*, it must be remembered that non-deficient emphysema and COPD still develops in the presence of “normal” physiological concentrations of AAT. This is because proteinase release by migrating neutrophils will always exceed the normal concentrations of AAT in the immediate environment of degranulation. Whether higher concentrations of AAT achieved in AATD by augmentation will restore this balance to a more favourable anti-proteinase state remains to be proven. However, in patients on conventional dose augmentation (60 mg·kg^−1^), doubling the dose does partly offset the inflammatory state and specifically reduces evidence of NE proteolytic damage [59], suggesting a dose-dependent benefit. A trial of double-dose AAT augmentation is currently being conducted by Grifols (SPARTA trial), although the outcome will not be known for 3 more years.

Secondly, recent studies have indicated that the pathophysiology of emphysema in non-deficient COPD is *via* initial loss of the small airways. This has also been implicated in AATD [13] and may reflect a different enzyme**/**cytokine pathway [60]. Without further study and a curative strategy for non-deficient emphysema, the concept and mechanism of such a pathway will remain speculative.

Thirdly, the proinflammatory effect of the Z protein and its polymers has been recognised but largely neglected in the aim to increase normal AAT. Heterozygotes (PiSZ and PiMZ) have not been considered to be at increased risk of developing COPD. However, recent careful studies of smoking and non-smoking non-index heterozygote and normal (PiMM) siblings has cast a different light on this issue. Smoking heterozygote siblings have an increased likelihood of developing COPD compared to their smoking PiMM siblings, despite AAT levels that exceed the putative protective threshold [6, 7]. This risk was not seen in non-smoking heterozygote siblings, whereas non-smoking PiZZ patients do have an increased risk for developing COPD. This raises the possibility that it is a combination of low protective AAT and polymers that have a proinflammatory effect. Indeed, cigarette smoke not only increases polymer formation [61] but associated oxidants also partly inactivate AAT as an elastase inhibitor [62], which probably explains increased risk in smoking PiZZ patients [63] and possibly an increased risk in PiMZ and PiSZ smokers [64].

Therefore, it could be argued that augmentation therapy converts a PiZZ homozygote to an PiM/PiZZ hybrid with an anti-inflammatory (M) and proinflammatory (ZZ) imbalance perpetuating some disease progression. Although this may explain the failure of augmentation to halt disease progression, it does not explain why only a proportion of PiZZ never-smokers develop significant disease, and further studies are clearly indicated to determine the potential reasons.

Such an issue would not apply to AAT null/null patients who have lower plasma and (by inference) lung levels of AAT, associated with more severe lung function impairment than ZZ individuals [65]. The lack of the proinflammatory Z protein may therefore increase the efficacy of augmentation, although the greater rarity of such patients presents an even greater challenge for such studies

Fourthly dosage based on patient weight may not always maintain AAT levels above the putative “protective” threshold due to variable individual metabolism and especially frequency of treatment. This however can be potentially overcome by measuring pre-dose AAT blood levels and adjusting the dose accordingly. These and several other issues are dealt with in detail in several recent publications on augmentation therapy [66, 67].

## The potential explanations for the missing PiZZ patients

In most cohort studies there are AATD subjects who appear healthy. This may reflect acquisition bias through family screening rather than case finding through identification from the presence of symptomatic disease. However, there have been multiple studies investigating the prevalence of AAT phenotypes in worldwide populations and hence projecting the number of individuals likely to be affected with severe AATD. These consistently suggest that the majority of PiZZ individuals have not been diagnosed. This certainly seems to be the case considering genetically based estimates, but there are two steps to diagnosis, namely confirmation of a relevant disease known to be associated with AATD and the requesting of an appropriate genetic test.

The most comprehensive study enabling this to be clarified was the report of the UK biobank in 2020 [68]. Of ∼460 000 participants of European ancestry (median age 58 years), 140 were the PiZZ genotype of whom only nine had been diagnosed. The data suggested a total UK population of 17 439 PiZZ individuals and an odds ratio of 8.8 for developing COPD and 7.3 for developing bronchiectasis. Of the patients analysed in the biobank data base, 17 790 had COPD of whom only 31 were the PiZZ genotype. The authors concluded that, even in patients with COPD, 77% of PiZZ individuals remain undiagnosed reflecting either poor awareness of the association, availability of testing and the possible contention that the lack of a putative “effective treatment” undermines the clinical need for testing. Alternatively, the undiagnosed cases may just be healthy, and this may be an issue with underdiagnosis even in countries where augmentation is available and requires further exploration/education of both doctors and patients.

The biobank study, therefore, still spotlights the potential reason(s) for low patient identification rates, and without widespread genetic testing and long-term population follow-up, these may remain unresolved. However, the Swedish birth cohort study [21] continues to provide data suggesting that some never- or ex-smokers with the PiZZ genotype do have evidence of pulmonary disease consistent with COPD and emphysema by the fifth decade. Similarly, retrograde analysis of lung function [20] suggests that, even though spirometry does not appear to deviate from normal until the fifth decade, gas transfer does so in the late 3rd decade. Consequently, gas transfer could also be expected to be abnormal by the age of 40 in at least some individual patients in the Swedish cohort, and this has now been confirmed [21].

The above cohort data thus suggest that a proportion of PiZZ patients do not or will not develop COPD, as shown in figure 2a, which contains data derived from the UK ADAPT (Antitrypsin Deficiency and Programme for Treatment) data base. Whilst this may potentially be subject to acquisition bias, ∼25–30% of never-smokers show no progressive decline in lung function greater than that expected for age, sex and height [40]. The reason the remainder do show abnormal decline in the absence of smoking remains unknown. Perhaps by matching the individuals with other known genetic polymorphisms [69], such as the functional tumour necrosis factor-α polymorphism [70] and the MMP 9 polymorphism described earlier [17], an answer may become evident.

## The hope offered by the development of new treatments

60 years has increased our knowledge of the role of AAT and the pathophysiology of AATD. In recent years this has led to an increasing search for curative therapy including small molecular inhibitors of NE, modified versions of AAT, inhaled AAT and other inhibitors, gene silencing, gene modification, modifying protein folding and NE production blockers amongst others. Whereas all these strategies may have benefit and logic behind their development, the biggest challenge remains the development of specific “proof of concept” phase 2 studies (to confirm that the agent acts appropriately on the strategic pathway) and then delivery of a powered phase 3 clinical trial with acceptable outcome(s.)

This latter step is currently the major challenge. Not only is the number of patients available and not on augmentation therapy low, but also the length of study that would be required to demonstrate a clinical benefit is unfeasible if physiological and health status stabilisation continue to be the only outcomes accepted by licencing and healthcare bodies.

**FIGURE 5:**
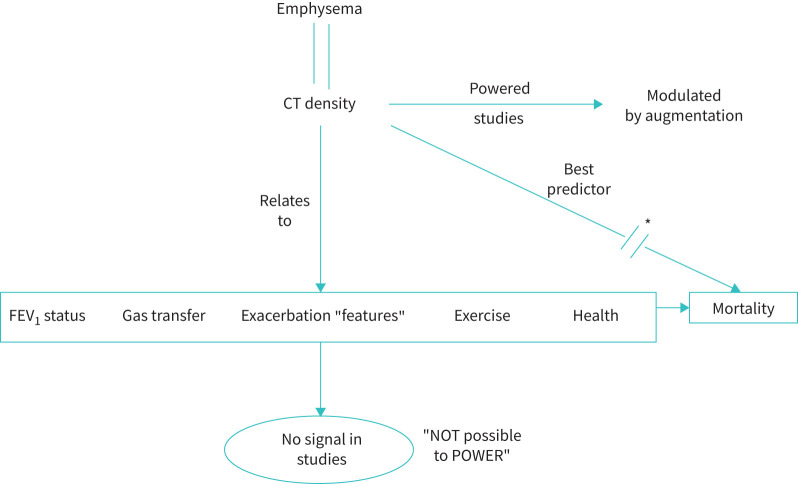
The clinical trials impasse. α_1_-Antitrypsin deficiency (AATD) is an emphysema-dominant type of COPD which can be specifically delineated and quantified by lung density using computed tomography (CT). Studies have shown that density relates to severity of impairment and decline of forced expiratory volume in 1 s (FEV_1_), impairment of gas transfer, the features of exacerbations and relates to both exercise capacity and health status measures in AATD. However, despite these relationships, CT densitometry remains the best predictor of mortality in AATD, and recent data confirm modulation of mortality by augmentation therapy (shown by the asterisk) [68]. The sensitivity of CT to emphysema change enables clinical trials to be powered demonstrating a positive effect on emphysema decline. However, none of the accepted “feel and function” parameters accepted as outcomes by drug advisory agencies can be powered into any study because of AATD rarity and those already on augmentation therapy providing a potential trials barrier for new therapies.

As stated above, CT densitometry is the most specific and sensitive marker of the emphysema process. Nevertheless, although it is clearly related to all of the relevant standard clinical measures of emphysema, the lack of sensitivity of these other measures to change [53] means studies cannot be powered for them. For the past 30 years, in recognition that FEV_1_ and health status are impractical outcomes, there has been a circuitous argument based on a request by the Food and Drug Administration (FDA) requiring data validating CT. Despite all the data now demonstrating the close relationships of CT to recognised and functional outcomes (as outlined in figure 5) and longitudinal data showing that lung density decline correlates with decline in FEV_1_ [42] and health status [53], the “validation” of CT is still judged by the FDA as incomplete.

CT densitometry remains an accepted outcome by experts and the European Medicines Agency (EMA), not least because it is the major predictor of mortality in AATD [54]. A recent matched open study has suggested that at least augmentation therapy is associated with reduced mortality [71] consistent with the findings of the data in the original NIH registry [39]. This may unblock the pathway to complete an observational circle enabling CT densitometry to become accepted as an outcome for many of these newer therapies. The alternative would be controlled trials of potential therapies in AATD patients with a high likelihood of future mortality, although it has to be accepted that many such patients are likely to be on augmentation therapy, again influencing the ability to power such studies.

## The patient perspective

The patient voice is of central importance in defining the unmet need especially in AATD and, irrespective of geographical location, there are some common themes that stand out [72, 73]. Improving knowledge of AATD, in particular in general practitioners, and access to AATD specialised centres are identified by patients as top priorities to ensure prompt diagnosis, the provision of reliable information following diagnosis and informed clinical management planning. Counselling and psychological support, as well as help in managing acute exacerbations and easy access to healthcare during these episodes, are also commonly reported to be important. It is unsurprising that patient perspective and experience differs according to whether they have access to augmentation therapy: in those countries where augmentation is reimbursed, patients have more frequent contact with healthcare and are better informed about their disease. Access to augmentation therapy is, understandably, a particular concern of patients in countries without reimbursement. Universal access to treatment, irrespective of country of residence, is considered to be just and fair, yet augmentation therapy is still unavailable in many European countries consequent to differing decisions towards reimbursement. It remains to be seen whether the European Union's plans to address such inequalities through legislation will achieve resolution of this disparity.

One of the most important additions to the understanding and management of AATD lung disease has been the international development of patient advocacy groups. These have facilitated clinical trial design and recruitment and, together with the academic scientists and clinicians, contributed to the development of research strategies and priorities [73]. Nevertheless, patient advocacy in AATD still lags in comparison with other rare diseases, and more has to be done by the AATD community to redress this.

## Conclusions

Understanding of the pathophysiology and natural history of AATD remains incomplete even after 60 years of study, which may seem surprising. This is, in part, because AATD is a “rare disease” but also because the introduction of augmentation therapy some 40 years ago has likely modified the natural history of the disease in those on treatment. Consequently, the number of patients available to enable observation of the “true natural history” is even lower, and attempts to monitor the disease in extensive detail and in the long term have been hampered. The data from the cohorts detailed above in this review are, therefore, all the more valuable. Although national and international registries have been established, low numbers, the impact of augmentation therapy in many countries and the cost of in-depth clinical phenotyping may leave many of the remaining uncertainties unanswered. These obstacles, combined with the failure to reach a consensus between experts and the FDA on suitable clinical outcomes, make the testing of efficacy of new therapies a continuing challenge. Nevertheless, scientifically and pharmacologically these issues are still of great pertinence to delivering optimal clinical care and are very much worth addressing.
